# Oral Microbiota and Type 2 Diabetes: Interactions, Potential Mechanisms, and Preventive Strategies

**DOI:** 10.3390/microorganisms14020336

**Published:** 2026-02-02

**Authors:** Zifu Ni, Zihan Ni, Yining Wang, Qi Wu, Zhenxi Yang, Yuqi Guo

**Affiliations:** 1 School of Bioengineering, Henan University of Technology, Zhengzhou 450001, China; 2 School of Stomatology, Henan Medical University, Xinxiang 453003, China

**Keywords:** oral microbiota, type 2 diabetes, periodontitis, interaction mechanism, prevention

## Abstract

The oral cavity harbors the second-largest and one of the most diverse microbial communities in the human body, playing a critical role in maintaining local and systemic health. Type 2 diabetes mellitus (T2DM), a chronic metabolic disease accounting for nearly 90% of all diabetes cases, has shown rapidly increasing global prevalence. Growing clinical and experimental evidence indicates a strong bidirectional relationship between oral microbiota dysbiosis and T2DM. Imbalanced oral microbial communities can contribute to systemic inflammation, insulin resistance, and metabolic dysregulation, while hyperglycemia and impaired immunity in T2DM promote oral diseases such as periodontitis, xerostomia, and mucosal infections. This review summarizes current research on the interactions between oral microbiota and T2DM, highlighting their clinical correlations, underlying mechanisms, and mutual influences on inflammation, microbial composition, and metabolic pathways. We also discuss emerging strategies for T2DM prevention and management through oral microbiota modulation. These insights may provide new perspectives for early diagnosis, targeted intervention, and integrative management of T2DM.

## 1. Introduction

The oral cavity is an important open organ of the human body and the initial segment of the digestive tract. It is primarily composed of the lips, cheeks, hard palate, soft palate, and other structures. As a major ecological niche for microbial colonization and survival, the oral cavity harbors a highly diverse microbial community, with more than 700 identified bacterial species and over 100 fungal species, as well as various archaea and viruses [1]. These microorganisms are not evenly distributed within the oral environment; instead, they cluster according to their specific growth characteristics and the distinct physiological conditions of different oral sites. Thus, areas such as the teeth, gingiva, gingival sulcus, tongue, lips, hard palate, soft palate, and even dental materials used in rehabilitation (including prostheses and implants) serve as colonization sites for microorganisms [2]. Through long-term interactions with the host, these microorganisms have formed a stable symbiotic relationship. Under the consistent supply of nutrients such as food debris in the oral cavity and proteins in bodily fluids, the composition and abundance of oral microbial communities remain in dynamic equilibrium [3]. These microorganisms not only share physical space with the host but also develop extensive and persistent biofilm systems, which play crucial roles in promoting the normal development of tissue structure and function, as well as preventing the colonization of pathogenic microorganisms [4]. In addition, the oral microbiota is closely associated with the gut microbiota. Approximately 10^11^ microorganisms migrate daily from the oral cavity to the stomach, and in human microbiome surveys, more than 45% of participants exhibit similar microbial profiles in both the oral cavity and feces [5]. During their growth and reproduction, these microorganisms continuously secrete a wide array of metabolites, which are also of great importance to human physiological functions and overall health.

**Figure 1:**
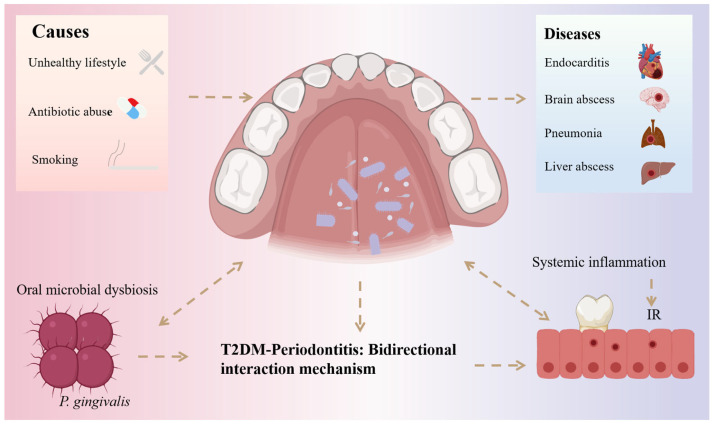
Bidirectional interplay between the oral microbiota and T2DM. Dashed arrows indicate relationships supported by human or animal studies. The figure highlights potential pathways through which oral microbial alterations may influence T2DM progression and vice versa, without implying definitive causality.

Under certain special conditions or environmental influences, such as poor lifestyle habits, overuse of antibiotics, and smoking, the oral microbiota can become imbalanced, thereby increasing the risk of recurrent aphthous ulcers, periodontitis, dental caries, oral cancer, and other oral diseases (Figure 1) [6,7,8]. In addition, dysbiosis of the oral microbiota can have significant impacts on systemic health. Damage to the oral mucosa allows bacteria to enter the bloodstream, leading to endocarditis, brain abscesses, pneumonia, and liver abscesses [9,10]. Epidemiological studies have shown that the oral microbiota is a risk factor for the onset and progression of atherosclerosis [11]. Fusobacterium nucleatum, a member of the oral microbiota, can indirectly promote tumor formation, and abnormal fluctuations in its abundance may serve as a potential marker for colorectal cancer development [12]. In recent years, with the steadily rising incidence and prevalence of diabetes, increasing attention has been directed toward identifying microbial and inflammatory factors that may interact with host metabolism. Within this context, the relationship between the oral microbiota and type 2 diabetes mellitus (T2DM) has emerged as an area of active investigation. Importantly, existing evidence reflects a complex interplay of bidirectional associations influenced by periodontal status, metabolic control, lifestyle factors, and medication use, rather than a simple unidirectional causal pathway. Collectively, these findings highlight the oral microbiota as a potential contributor to systemic health and disease within a multifactorial framework.

T2DM is one of the most common clinical forms of diabetes, accounting for more than 85% of all cases [13]. This condition is primarily caused by insulin resistance (IR) or dysfunction of pancreatic β-cells [14]. In addition, low-grade chronic inflammation is considered an important contributing factor in the pathogenesis of T2DM and is involved in the development of IR [15]. In recent years, studies have revealed that T2DM is closely associated with a variety of oral diseases, including xerostomia, periodontitis, gingivitis, taste disorders, impaired wound healing, and oral mucosal diseases, all of which severely affect patients’quality of life [16,17,18]. Among these conditions, periodontitis is a chronic inflammatory disease mainly induced by dental plaque biofilms [19]. As shown in Figure 1, several bacterial species, including *Porphyromonas gingivalis*, *Tannerella forsythia*, and *Treponema denticola,* are strongly associated with its onset and progression [20]. Numerous studies have demonstrated a bidirectional relationship between T2DM and periodontitis [17,18,19,20]. Individuals with T2DM are more susceptible to developing periodontitis, potentially due to hyperglycemia-induced elevations in pro-inflammatory cytokines and impaired immune responses, which disrupt the oral microbiota and ultimately trigger periodontal inflammation [21]. Conversely, patients with periodontitis also exhibit an increased risk of developing T2DM. On the one hand, periodontal disease adversely affects glycemic control and exacerbates complications in T2DM patients [22]. On the other hand, specific periodontal microorganisms have been shown to be associated with T2DM; in addition to the bacteria mentioned above, subgingival microbes such as *Aggregatibacter*, *Prevotella*, and *Neisseria* exhibit significant differences in abundance between T2DM and non-type 2 diabetic patients [23]. Periodontal therapy has been shown to improve glycemic control and reduce systemic inflammation in T2DM patients with coexisting periodontitis, while also exerting selective influences on periodontal microbial communities at the species level [24]. Collectively, these findings provide new insights into the pathogenesis of T2DM and offer promising directions for the development of novel therapeutic strategies.

With the continuous increase in the prevalence of T2DM, it has become one of the fastest-growing global public health emergencies [25]. Early prediction, diagnosis, and timely treatment of T2DM have therefore become urgent priorities for research. T2DM can cause subtle changes in the abundance of certain oral bacteria, including key pathogenic species, thereby triggering inflammatory cascade reactions that ultimately result in oral microbial dysbiosis and periodontal disease [26]. Once the homeostasis of the oral microbiota is disrupted, the decline in commensal bacteria and the proliferation of pathogenic species can destabilize the healthy microenvironment of the oral cavity. Certain pathogenic bacteria within the host microenvironment may elevate local or systemic inflammation by releasing virulence factors such as lipopolysaccharides (LPS), leading to chronic inflammation and further influencing the progression of T2DM [27]. Thus, a specific association exists between the oral microbiota and T2DM. However, given the complexity and spatiotemporal characteristics of oral microbial alterations in T2DM patients, a systematic review is needed to clarify the current state of research. In this context, the present review summarizes recent progress in studies concerning the relationship between the oral microbiota and T2DM. It outlines and discusses their interactions, influencing factors, and biological effects, with the aim of providing valuable insights for research on oral microbiota, T2DM, and oral diseases, as well as offering new perspectives for the prevention and treatment of T2DM.

In this review, relevant articles were identified through searches in PubMed, Web of Science, and Scopus using keywords including “oral microbiota,” “periodontitis,” and “T2DM.” Priority was given to recent systematic reviews, longitudinal cohort studies, interventional periodontal therapy trials, and multi-omics sequencing studies. Quantitative statements, such as relative risk estimates and percentage changes, are explicitly linked to their original sources, and the evidence level is clearly indicated, distinguishing meta-analyses, cohort studies, randomized clinical trials, and mechanistic studies. Furthermore, in this review, the term “oral microbiota” is used as an integrative concept encompassing microbial communities sampled from multiple oral niches, including saliva, tongue, supragingival plaque, and subgingival plaque. However, many studies in the current literature do not consistently report or stratify sampling sites. Consequently, site-specific conclusions cannot always be drawn, and statements in this review reflect the level of resolution supported by the available evidence.

## 2. Interactions Between the Oral Microbiota and T2DM

### 2.1. Oral Microbial Dysbiosis and Its Association with T2DM

In the complex environment of the oral cavity, diverse microorganisms form a species-rich and heterogeneous ecological system. This system is occupied by different microbial species depending on local environmental conditions, thereby maintaining a relatively stable equilibrium. Among these microorganisms, bacteria represent the most abundant group and include members of the *Firmicutes*, *Spirochaetes*, *Proteobacteria*, and *Actinobacteria phyla* [28,29]. The oral cavity also contains a variety of fungi, among which *Candida* species are the most common [30]. When the balance of the oral microbiota is disrupted, the dysbiotic microbial community can contribute to the development of various inflammatory diseases by excessively accumulating virulence factors and regulating inflammasome-targeted pathways [31,32]. In recent years, high-throughput sequencing analyses of 16S ribosomal RNA (16S rRNA) genes from the oral microbiota of T2DM patients and healthy individuals have revealed a close association between the oral microbiome and both the onset and progression of T2DM [33]. The colonization levels of certain oral bacteria, including *Aggregatibacter actinomycetemcomitans*, *Porphyromonas gingivalis*, and *Tannerella forsythia,* show a positive correlation with the incidence of prediabetes [34]. Notably, the relative abundances of these bacteria are also significantly higher in individuals with periodontitis compared with healthy subjects. In addition, the abundance of Actinobacteria is markedly lower in T2DM patients than in healthy controls. Within this phylum, five families and seven genera have been identified, most of which appear in reduced levels among individuals with T2DM [35]. This observation is consistent with findings from microbiological surveys of patients with periodontitis. Furthermore, genera such as *Actinomyces* and *Atopobium* are associated with reduced T2DM risk, by 66% and 72%, respectively, with *p*-values of 8.9 × 10^−3^ and 7.4 × 10^−3^ [36]. These bacteria are also related to the prevalence of obesity and have been shown to influence blood glucose levels. Chronic hyperglycemia, accompanied by elevated levels of infection- and inflammation-induced advanced glycation end products (AGEs), may help explain the clinical manifestations of periodontal disease in diabetic patients [37]. These factors play significant roles in the pathogenesis of T2DM and underscore the importance of oral microbial dysbiosis in the disease process.

Recent studies have shown that oral bacteria can accumulate in the gut of patients with various diseases, leading to activation of the intestinal immune system and subsequent chronic inflammation [38]. Although it has not yet been fully established how oral microorganisms reach organs of the digestive system, evidence increasingly supports that the invasion of oral pathogens can disrupt gut microbial homeostasis and contribute to systemic diseases [36]. *Porphyromonas gingivalis* has been shown to translocate from the oral cavity to the gut in several conditions, including colorectal cancer, inflammatory bowel disease (IBD), and T2DM [35]. Increased intestinal permeability to bacterial LPS is an important factor that triggers systemic inflammatory responses [39]. Short-chain fatty acids produced by gut microbes play critical roles in maintaining intestinal mucosal integrity [33]. However, in T2DM, the production of butyrate is significantly reduced, leading to increased gut permeability [40].

### 2.2. Effects of T2DM on Oral Microbial Community Structure and Microenvironment

#### 2.2.1. Effects of T2DM on Oral Microbial Diversity

The impact of T2DM on the oral microbiota began to attract attention after it was recognized that T2DM is a major risk factor for severe and progressive periodontitis, a condition characterized by infection- or lesion-induced destruction of supporting tissues and alveolar bone [23]. Elevated glucose levels in the gingival crevicular fluid of T2DM patients may promote the growth of certain bacterial species, thereby influencing the diversity of the oral microbiota [25]. Some studies have reported a relative increase in the abundance of certain Gram-positive taxa in specific oral niches of individuals with T2DM. However, taxa- and niche-specific alterations in the oral microbiota, with considerable heterogeneity across oral sites and study designs. Increased production of interleukin-17 (IL-17) in T2DM may further alter the oral microbiota, making it more pathogenic [41]. In turn, the oral microbiota influences systemic health by suppressing potential pathogens, modulating immune responses, and contributing to nutrient absorption and metabolism. In recent years, deep sequencing, metagenomics, and other advanced technologies have revealed marked differences in the oral microbial communities of T2DM patients compared with healthy individuals [42]. Poor glycemic control in T2DM patients has been associated with reduced oral microbial diversity, which may be linked to decreased levels of butyrate-producing bacteria and increased levels of taxa associated with propionate and succinate production, potentially elevating the abundance of opportunistic pathogens [43]. Nevertheless, some studies suggest that blood glucose levels have only a minimal impact on the composition of the oral microbiota [44]. Compared with individuals with normal glycemia, both T2DM and prediabetic patients exhibit reduced oral microbial diversity. These findings indicate that T2DM may induce alterations in the oral microbial community. However, different bacterial taxa may exhibit distinct patterns of change. The inconsistencies among studies investigating the effects of T2DM on oral microbial diversity may be attributed to factors such as participant age, sampling methods, and sampling sites [45]. Additionally, when considering type 1 diabetes (T1DM), emerging evidence suggests both shared and distinct patterns of oral microbial dysbiosis [46]. While reduced microbial diversity and increased abundance of opportunistic pathogens are common features in both T1DM and T2DM, differences exist in the prevalence of specific taxa and the magnitude of inflammatory responses [47]. This comparison provides a broader perspective on diabetes-associated oral microbiota alterations and underscores the importance of considering diabetes type when evaluating microbial and inflammatory changes.

#### 2.2.2. Effects of T2DM on the Abundance of Oral Microbiota

The dominance and composition of the oral microbiota undergo substantial alterations, which are closely associated with inflammation, osteoclastogenesis, and periodontal bone loss [35]. In general, T2DM tends to increase the overall abundance of oral microorganisms. Compared with healthy individuals whose oral microbiota is dominated by species such as *Clostridium acetobutylicum*, hemolytic *Streptococcus*, and members of the *Staphylococcus* genus, patients with T2DM exhibit significantly elevated levels of certain oral pathogens, particularly *Porphyromonas gingivalis* [43]. Poor glycemic control in T2DM has been linked to increased numbers of red complex bacteria within subgingival biofilms. The red complex refers to a group of strictly anaerobic periodontal pathogens, including *Porphyromonas gingivalis*, *Tannerella forsythia*, and *Treponema denticola*. These species frequently co-occur in periodontal pockets, exhibit strong synergistic interactions, and are strongly associated with advanced forms of periodontitis, including deep periodontal pockets, clinical attachment loss, and alveolar bone resorption [42]. Moreover, although both T2DM and non-type 2 diabetic individuals show elevated levels of *Actinomyces*, *Capnocytophaga*, *Eikenella corrodens*, *Campylobacter rectus*, *Fusobacterium nucleatum*, *P. gingivalis*, and *Prevotella intermedia*, the overall bacterial load tends to be slightly higher in T2DM patients [46]. Environmental and geographical factors also influence the quantity of oral microbiota. For instance, in patients with T2DM in Pakistan, the phylum Firmicutes and its associated genera such as Prevotella and Leptotrichia both acidogenic bacteria are more abundant than in healthy controls [47]. Additionally, species of *Streptococcus* and *Lactobacillus* isolated from supragingival plaque are reported to be more numerous in Thai individuals with T2DM compared with those without the disease [48].

Additionally, some studies have revealed that T2DM may be negatively associated with the levels of certain oral microbial taxa, such as *Proteobacteria* and *Bifidobacteria* [49]. However, other members of the oral microbiota may remain largely unaffected by blood glucose levels. In T2DM patients, certain oral microorganisms may be diminished or even absent. For instance, *Proteobacteria* are often undetectable in T2DM patients but are commonly present in healthy individuals [46]. Several studies also suggest that the abundance of periodontal oral microbiota in T2DM patients may indeed differ from that of individuals with normal glycemia, likely because the composition of many oral microbial taxa changes in response to fluctuations in blood glucose levels. Among 44 oral microbial taxa examined in diabetic patients, the abundance of 27 taxa increases with rising blood glucose, whereas 17 taxa increase as blood glucose decreases [50].

#### 2.2.3. Effects of T2DM on the Oral Microenvironment

Saliva provides a suitable environment for the survival of various oral microbial communities, and its ionic, gaseous, and organic components may play regulatory roles [51]. T2DM can alter the composition of saliva and gingival crevicular fluid (GCF), including increased glucose levels and elevated concentrations of various inflammatory mediators [48]. In T2DM patients, rising blood glucose levels are accompanied by corresponding increases in glucose concentrations in saliva and GCF [52]. On one hand, this high-sugar environment supplies abundant nutrients that promote bacterial growth; on the other hand, certain oral pathogens, such as *Streptococcus mutans*, can metabolize these sugars to produce acidic byproducts, lowering the pH around dental plaque and providing a growth advantage for acid-tolerant species [51]. In patients with poorly controlled T2DM, carbohydrate-metabolizing bacteria, including *Streptococcus*, *Prevotella*, and *Veillonella* species become more abundant [52]. This not only alters the diversity of the oral microbiota but also increases the risk of dental caries. Additionally, T2DM patients often exhibit reduced salivary secretion, frequently accompanied by xerostomia [53]. These conditions can promote colonization by various *Candida* species, further disrupting microbial diversity in the oral cavity. Elevated blood glucose may also increase oral acidity, affecting microbial balance [53]. Furthermore, T2DM can alter other microenvironmental factors such as redox potential and the availability and concentration of nutrients, thereby influencing bacterial growth and development [54].

## 3. Mechanistic Links Between the Oral Microbiota and T2DM

### 3.1. Impact of Periodontal Dysbiosis on Glycemic Control and T2DM Progression

Individuals with periodontitis have a fourfold higher risk of developing T2DM compared with healthy controls, and the risk is further increased by 53% in patients with severe periodontitis [55]. Among non-diabetic individuals, those with periodontitis exhibit significantly higher fasting blood glucose, glycated hemoglobin A1c (HbA1c), and insulin resistance indices compared with healthy controls, suggesting that periodontitis may contribute to an elevated risk of T2DM [56]. The presence of periodontitis may also interfere with glycemic control in T2DM patients. Clinical studies consistently indicate that individuals with both T2DM and periodontitis generally demonstrate poorer glycemic management than those with well-maintained periodontal health [57]. Maintaining stable blood glucose levels is crucial for T2DM patients, as it directly impacts disease progression and clinical outcomes [55]. Poor glycemic control can accelerate the onset of T2DM-related complications, including diabetic retinopathy, diabetic nephropathy, diabetic foot, and cardiovascular disease [58]. Furthermore, studies have shown that T2DM patients with concurrent periodontitis have a significantly higher risk of developing these complications. Collectively, these findings indicate that periodontitis not only poses a challenge to glycemic management in diabetic patients but is also closely associated with the progression of T2DM-related complications.

**Figure 2:**
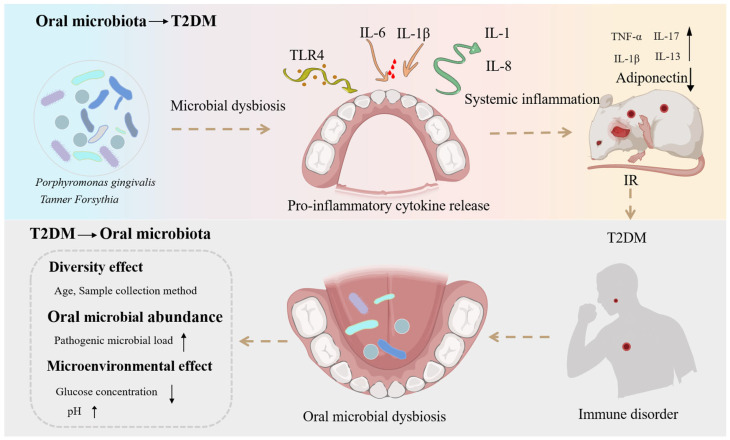
Mechanistic investigation of the interplay between the oral microbiota and T2DM. Solid arrows indicate pathways supported by human or animal studies, whereas dashed arrows represent associative or indirect links. The figure illustrates potential interactions between oral microbial dysbiosis, systemic inflammation, and metabolic dysregulation, without implying definitive causality.

Considering the pivotal role of chronic inflammation in the pathogenesis of T2DM, bacterial components in the oral cavity play an important role in modulating host inflammatory responses. The oral pathogen *Porphyromonas gingivalis* produces LPS and fimbriae that can activate the nucleotide-binding oligomerization domain (NOD)-like receptor family, pyrin domain-containing 3 (NLRP3) inflammasome, leading to elevated serum levels of IL-6 and IL-1β and contributing to the progression of periodontitis [59]. Among the inflammasome family, NLRP3 has been studied most extensively and is implicated in both autoimmune and various non-autoimmune chronic diseases [60]. Upon stimulation by endogenous molecules or pathogen-associated molecular patterns, the NLRP3 inflammasome assembles and activates, promoting the maturation and secretion of pro-inflammatory cytokines [61]. The NLRP3/ASC-caspase-1 multiprotein complex has been shown to modulate innate immune responses in both T2DM and periodontitis [62]. Periodontitis and the associated bacterial burden may further upregulate NLRP3 levels in serum and saliva, thereby amplifying inflammatory processes [61]. In addition, the fimbriae of *P. gingivalis* can bind to Toll-like receptors, promoting the release of pro-inflammatory cytokines such as IL-1 and IL-8. As shown in Figure 2, its LPS can also induce the production of TNF-α, IL-1β, IL-17, IL-13, and interferon-γ in vivo in mice [63]. These inflammatory mediators not only exacerbate periodontal tissue inflammation but may also trigger systemic inflammatory responses, thereby aggravating insulin resistance [60]. These findings indicate that the oral microbiota can influence systemic inflammation and insulin resistance through multiple mechanisms, thereby contributing to the onset and progression of T2DM.

Periodontitis may influence glucose metabolism in humans through two primary pathways: the periodontal pocket–bloodstream route and the oral-to-gut microbial translocation pathway [61]. When the periodontal pocket is compromised, bacteria and their metabolites can penetrate the pocket wall and enter the bloodstream, triggering systemic inflammatory responses that may interfere with normal insulin function and destabilize blood glucose levels [64]. Simultaneously, oral microbiota associated with periodontitis may enter the digestive system via saliva, potentially disrupting gut microbial homeostasis and impairing intestinal barrier function, thereby exacerbating systemic inflammation and adversely affecting glucose metabolism (oral–gut axis) [65].

(1) Periodontal pocket–bloodstream pathway: Human observational and clinical evidence indicates that periodontitis is associated with a persistent low-grade systemic inflammatory state, potentially mediated by the anatomical and functional connection between periodontal pockets and the bloodstream [64]. In terms of ulcerated surface area, the total periodontal pocket area in patients with severe periodontitis can reach up to 72 cm^2^, providing a substantial potential reservoir for bacterial colonization [65]. Gram-negative bacteria are predominant within these pockets and are rich in endotoxins such as lipopolysaccharides (LPS) [62]. When the integrity of the pocket epithelium is compromised, bacterial components and endotoxins may gain access to the circulation, contributing to systemic inflammatory responses. From a frequency and exposure perspective, clinical observational studies have shown that patients with severe periodontitis frequently experience transient bacteremia and endotoxemia during routine oral hygiene practices, including tooth brushing and flossing [66]. Moreover, bacteremia levels induced by brushing have been reported to correlate positively with plaque accumulation and the severity of gingival inflammation [64]. Taken together, these human-based observations support an association between periodontitis and chronic, low-grade systemic inflammation via the periodontal pocket–bloodstream pathway, although direct causality cannot be fully established based on observational evidence alone.

(2) Oral–gut microbial translocation pathway: Observational studies have reported that saliva from patients with periodontitis contains significantly increased levels of putative periodontal pathogens [67], raising the possibility that oral bacteria may reach the gastrointestinal tract through swallowing. This hypothesis is supported primarily by experimental animal studies. For example, oral administration of Porphyromonas gingivalis in mice has been shown to alter gut microbial composition and is accompanied by systemic inflammation and insulin resistance [68]. Similarly, oral gavage of Aggregatibacter actinomycetemcomitans in murine models, under both normal and high-fat dietary conditions, results in impaired glucose tolerance, insulin resistance, and gut microbiota dysbiosis [69]. These mechanistic animal studies provide biological plausibility for an oral–gut microbial translocation pathway linking periodontitis-associated bacteria to metabolic disturbances. However, although impaired glucose metabolism and gut microbial alterations have been observed concurrently, direct evidence from human interventional studies demonstrating that modulation of gut microbiota mediates these metabolic effects remains limited [66]. The specific mechanisms by which periodontitis-associated oral bacteria interact with the gut microbiome, the key oral and intestinal taxa involved, and the extent to which these findings can be extrapolated to human T2DM require further investigation [68].

In summary, existing evidence from observational human studies and experimental animal models suggests that periodontitis may be linked to the onset, progression, and outcomes of T2DM through pathways involving bacterial translocation from periodontal pockets into the bloodstream, contributing to systemic inflammation, as well as the migration of oral bacteria to the gut via saliva, potentially leading to gut microbial dysbiosis, barrier dysfunction, and metabolic inflammation that may exacerbate insulin resistance.

### 3.2. Inflammation-Driven Oral Microbial Dysbiosis in T2DM

There exists a complex bidirectional interaction between T2DM and periodontitis, in which inflammatory processes play a central role [48]. In patients with T2DM, levels of pro-inflammatory cytokines released from periodontal tissues such as tumor necrosis factor-α (TNF-α) and interleukin-1β (IL-1β) are elevated [52]. The increased presence of these inflammatory mediators exacerbates periodontal tissue damage and creates a growth advantage for periodontitis-associated microbes, while simultaneously reducing the abundance of microbial species that maintain periodontal health [61]. Inflammation-induced tissue destruction releases peptides, collagen fragments, and iron-containing compounds, which may serve as nutrients for specific microorganisms [69]. Upon entering the gingival crevice, these components further promote the growth of iron-utilizing bacteria, such as *Porphyromonas gingivalis* [44]. This pathogen relies on the inflammatory environment for nutrient acquisition and can evade host immune clearance through complex mechanisms, thereby impacting both the composition and abundance of the oral microbiota [53]. Overall, the inflammatory responses induced by T2DM disrupt the homeostasis of the oral microbial community, providing increased resources for pathogenic bacteria, enhancing their virulence, and aggravating inflammation and periodontal tissue destruction, thereby creating a vicious cycle [70]. This phenomenon underscores the complexity of the reciprocal relationship between diabetes and oral diseases.

**Figure 3:**
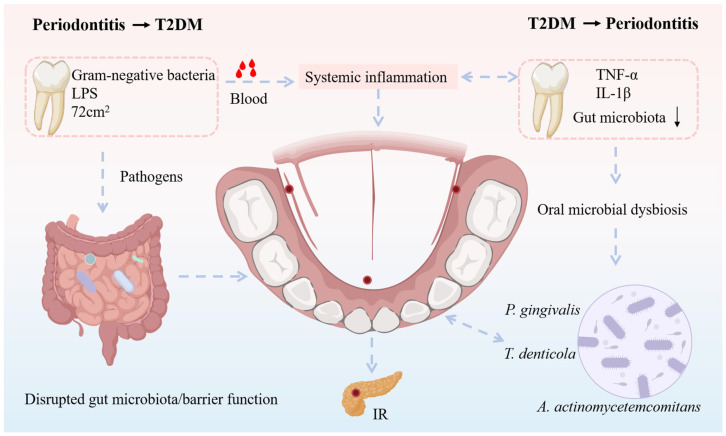
Oral dysbiosis drives systemic inflammation and insulin resistance through the periodontitis–T2DM interaction axis. Solid arrows indicate pathways supported by human or animal studies, whereas dashed arrows represent associative or indirect links. This figure illustrates possible interactions without implying definitive causality.

As shown in Figure 3, periodontitis is a chronic inflammatory disease primarily caused by the destruction of periodontal tissues by bacteria within dental plaque, which compromises the supporting structures of the teeth [60]. T2DM increases the risk, severity, and negatively impacts the prognosis of periodontal therapy. Recent data indicate that T2DM patients have a 1.58-fold higher risk of developing periodontitis compared with controls, and poor glycemic control further increases the risk of periodontitis by 34% [71]. Compared with healthy controls, T2DM patients exhibit poorer periodontal conditions, with an average increase in periodontal pocket depth of 0.61 mm [70]. Notably, good glycemic control does not appear to elevate the likelihood of periodontal disease, whereas poor glycemic control significantly increases this risk, potentially due to heightened systemic inflammatory responses under hyperglycemic conditions [68]. These findings suggest that inadequate glycemic management may promote the onset and progression of periodontitis.

T2DM may also influence the oral microbial community, altering its composition in ways that enhance the pathogenic potential of certain bacteria, thereby exacerbating periodontitis [38]. Clinical studies have observed that the subgingival microbiota of periodontitis patients with T2DM differs from that of non-diabetic patients [46]. Furthermore, glycemic control interventions may help restore balance within the oral microbiota. In the periodontal pockets of T2DM patients, increased levels of periodontopathogens including *Porphyromonas gingivalis*, *Tannerella denticola*, and *Aggregatibacter actinomycetemcomitans* have been detected, suggesting a potential association with the development of T2DM [55]. However, most current studies are cross-sectional, making it difficult to establish a causal relationship between T2DM and changes in the oral microbiota [72]. Longitudinal cohort studies are needed to further verify this hypothesis, and controlled animal experiments can provide preliminary mechanistic evidence. Experimental observations in T2DM mouse models have shown alterations in the oral microbiota, and microbial transplantation of these communities into germ-free mice induced periodontal inflammation and bone resorption, indicating that poorly controlled T2DM may increase the risk of periodontitis progression [73].

In T2DM patients with periodontitis and poor glycemic control, concentrations of IL-1β and IL-6 in periodontal tissues are significantly higher than in patients with periodontitis alone [67]. Poorly controlled T2DM exacerbates periodontal inflammation, with serum and gingival crevicular fluid levels of inflammatory markers such as TNF-α and IL-6 generally exceeding those observed in well-controlled T2DM patients or patients with periodontitis only [70]. These clinical findings suggest that T2DM may promote periodontitis by intensifying both systemic and localized inflammatory responses [68]. Animal studies corroborate these observations, consistently demonstrating that T2DM enhances inflammatory mediator activity within periodontal tissues. In experimental models, periodontal tissues of T2DM animals show significantly elevated levels of IL-1β, TNF-α, IL-6, and innate immune receptors such as TLR2 and TLR4, reflecting an overactivated immune response [74]. This excessive inflammation not only exacerbates periodontal tissue inflammation and degradation but also promotes osteoclastogenesis and bone resorption, resulting in further periodontal destruction. Collectively, these findings indicate that T2DM may accelerate the progression of periodontitis by elevating inflammatory cytokine levels in periodontal tissues.

## 4. Preventive Strategies Targeting the Oral Microbiota in Type 2 Diabetes Mellitus

Dysbiosis of the oral microbiota can trigger chronic inflammation and persistent infection, establishing immune memory within immune cells and leading to an exaggerated response to inflammatory and bacterial signals [34]. This increases the risk of multiple diseases, includingT2DM, and accelerates disease progression. Meanwhile, T2DM patients who develop oral complications often experience greater difficulty in glycemic control, accompanied by alterations in the composition and function of the gut microbiota [47]. Therefore, maintaining the ecological balance of the oral microbiota, monitoring its dynamic changes, and implementing timely, targeted preventive strategies can exert beneficial effects on disease management in individuals with T2DM.

### 4.1. Oral Microbiota as Biomarkers for Type 2 Diabetes Mellitus

Disruption of oral microbiota homeostasis has been reported as a potential early indicator of T2DM development, and several microbial taxa have been statistically associated with glycemic traits or disease onset in individual studies [75]. Specifically, nine oral bacterial genera have been identified as predictors of blood glucose fluctuations based on statistically significant associations or predictive modeling approaches. For example, elevated blood glucose levels are associated with increases in *Atopobium* spp. and *Stomatobaculum* sp. (*p* = 0.005), whereas decreases in blood glucose are correlated with the abundance of *Leptotrichia* spp. (*p* = 0.0012) [76]. In addition, multiple species have been reported to show statistically significant associations with incident diabetes or predictive performance in classification models, including *Eubacterium sulci*, *Lactiplantibacillus paraplantarum*, *Megasphaera micronuciformis*, *Acinetobacter nosocomialis*, *Solobacterium moorei*, *Limosilactobacillus mucosae*, *Streptococcus acidominimus*, *Phocaeicola abscessus*, *Klebsiella oxytoca*, *Shuttleworthella satelles*, *Streptococcus salivarius*, *Enterobacter cloacae*, *Staphylococcus aureus*, *Prevotella oralis*, *Pseudomonas aeruginosa*, *Aggregatibacter actinomycetemcomitans*, and members of the phylum *Fusobacteria* [77]. However, due to heterogeneity in study designs, population characteristics, sample collection sites, and statistical methodologies, direct comparison of *p*-values or effect sizes across studies is not feasible. Potential issues such as cohort effects, batch effects, overfitting in predictive models, and limited external validation further constrain the reliability of these findings. Therefore, these taxa should be regarded as candidate or exploratory biomarkers rather than validated diagnostic markers. Monitoring and analyzing the oral microbiota can help identify individuals at high risk for T2DM, enabling early preventive interventions. Moreover, due to differences in lifestyle, environment, and habits, the composition and functional profiles of the oral microbiota vary substantially among individuals. Metagenomic sequencing combined with untargeted metabolomic analysis can reveal correlations between oral microbes and their metabolites, thereby providing a basis for identifying early diagnostic biomarkers for oral complications in T2DM [78]. In addition, tailoring preventive measures such as dietary adjustments and targeted oral hygiene interventions to an individual’s oral microbiota profile may further enhance prevention and early-warning strategies for T2DM-related oral diseases.

### 4.2. Modulating the Oral Microbiota to Suppress Inflammatory Responses

**Figure 4:**
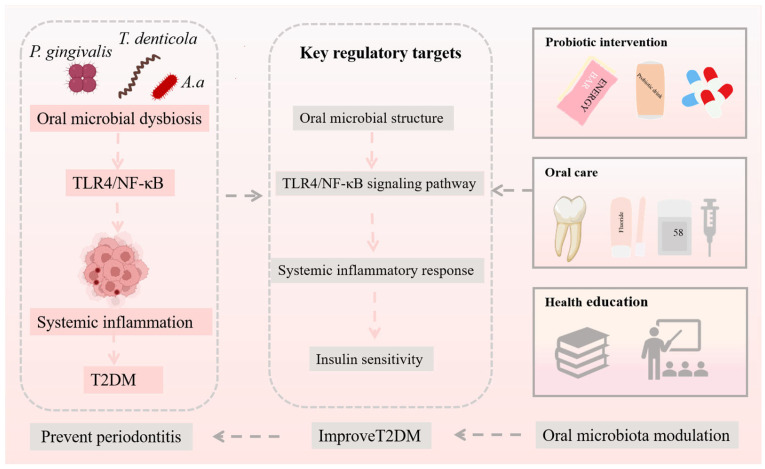
Preventive strategies of the oral microbiota against T2DM.

Alterations in the oral microenvironment of patients with T2DM increase their susceptibility to oral infections, particularly periodontitis (Figure 4) [79]. Such infections, accompanied by both local and systemic inflammatory responses, can adversely affect glycemic control. Periodontitis is one of the major complications of T2DM, and a bidirectional relationship exists between the progression of diabetes and periodontitis [76]. Clinically validated strategies for managing periodontal health in T2DM patients include maintaining oral hygiene and professional periodontal therapy. Periodontal treatment, including scaling and root planing (SRP), has been shown to improve periodontal parameters and reduce systemic inflammatory markers, and can modestly improve glycemic control in diabetic patients [80,81,82]. Adjunctive use of antimicrobial mouthwashes or antibiotics such as amoxicillin and metronidazole may further reduce periodontitis-associated bacteria, though these interventions should be carefully considered due to risks of resistance and systemic side effects [81,82]. Emerging or experimental microbiota-targeted approaches, including probiotic supplementation, synbiotics, and oral microbiota transplantation, have been investigated in small studies or preclinical models [83,84,85,86]. Oral probiotics, particularly *Lactobacillus* and *Bifidobacterium species*, have shown potential to inhibit periodontopathogenic bacteria and reduce local inflammation. Synbiotics may further support beneficial oral microbial communities. However, the clinical efficacy, safety, long-term effects, regulatory considerations, and ethical implications of these strategies remain insufficiently studied, and no high-quality clinical trials specifically targeting oral microbiota to improve metabolic outcomes in T2DM currently exist. Therefore, these approaches should be considered experimental, and their application should be cautious and evidence-based. Collectively, currently recommended measures with demonstrated clinical relevance focus on maintaining oral hygiene, professional periodontal therapy, and optimal glycemic control, which together can improve periodontal health and attenuate systemic inflammation in T2DM patients. Experimental microbiota-targeted interventions may hold future promise but require rigorous evaluation before routine clinical implementation.

### 4.3. Modulating Metabolic Processes Through the Oral Microbiota

The oral microbiota can metabolize nutrients and produce a variety of metabolites that exert significant effects on host metabolic processes. Through the fermentation of carbohydrates and amino acids, oral microbes generate short-chain fatty acids (SCFAs), such as butyrate, acetate, and propionate [87]. These SCFAs possess antimicrobial properties and play regulatory roles in host immune and metabolic pathways [88]. Butyrate, for instance, can stimulate the secretion of glucagon-like peptide-1 (GLP-1) and peptide YY (PYY), thereby enhancing insulin secretion and reducing appetite [89]. Nitrate-reducing oral bacteria can convert nitrate into nitrite, which is subsequently transformed into nitric oxide, a key signaling molecule involved in cardiovascular and metabolic regulation. Nitric oxide has been associated with blood pressure reduction, improved endothelial function, and antidiabetic effects [90]. Similarly, probiotics and prebiotics may further influence host metabolic processes by modulating the metabolic activity of the oral microbiota [91]. Certain probiotic strains are capable of enhancing the production of beneficial metabolites, including short-chain fatty acids and nitric oxide–related intermediates, thereby contributing to improved insulin sensitivity and metabolic homeostasis [92]. Prebiotics, by selectively promoting the growth and activity of beneficial oral bacteria, may further amplify these metabolic effects. There is substantial interplay between the oral and gut microbiota. Oral bacteria can enter the gastrointestinal tract through swallowing, thereby altering the composition and function of the gut microbiome [93]. Considering the critical role of gut microbiota in the onset and progression of T2DM, particularly through its regulation of host metabolism, immune responses, and inflammation, maintaining oral microbial balance may indirectly modulate the gut microbiome and contribute to the prevention of T2DM.

## 5. Conclusions

T2DM and oral health—particularly periodontitis—are closely interconnected through a complex bidirectional relationship in which the oral microbiota plays a central role. Dysbiosis of the oral microbial community can promote systemic inflammation, impair metabolic homeostasis, and contribute to insulin resistance, whereas hyperglycemia and immune dysregulation in T2DM alter the oral microenvironment, favoring the proliferation of pathogenic bacteria and accelerating periodontal tissue destruction. Although substantial progress has been made in characterizing these interactions, many mechanisms underlying oral–systemic crosstalk in T2DM remain insufficiently understood. A deeper understanding of the oral microbiota’s role in T2DM may ultimately enable the development of novel preventive, diagnostic, and therapeutic tools, supporting a more integrated and personalized approach to managing this increasingly prevalent disease.

## Data Availability

No new data were created or analyzed in this study.
